# Leading With Callings: Effects of Leader’s Calling on Followers’ Team Commitment, Voice Behavior, and Job Performance

**DOI:** 10.3389/fpsyg.2018.01706

**Published:** 2018-09-12

**Authors:** Jiyoung Park, Kyoungsu Lee, Jung In Lim, Young Woo Sohn

**Affiliations:** Department of Psychology, Yonsei University, Seoul, South Korea

**Keywords:** leader’s calling, transformational leadership, team commitment, voice behavior, job performance

## Abstract

Viewing work as a calling has been considered to be beneficial to individuals and organizations. However, research to date has largely focused on the effects of individuals’ own callings on themselves, leaving the effects of one’s calling on others unexplored. Based on research that demonstrates prevalent effects of callings and leader’s influences on followers at work, we assumed that leader calling might have positive effects on followers’ outcomes. Specifically, we hypothesized that the extent to which leaders view their work as a calling have positive influences on followers’ team commitment, leader-rated voice behavior, and job performance. We also examined a mediating effect of transformational leadership on the relations between leader’s calling and the three follower’s outcomes. Using data on 284 leader-follower pairs from the South Korean Air Force, we found that leader’s calling was positively associated with followers’ team commitment, voice behaviors, and job performance. The effects of leader’s calling on follower commitment and voice behavior were partly accounted for by follower perceptions of transformational leadership. However, a mediating role of transformational leadership on the link between leader’s calling and job performance was not supported. The implications of these findings are discussed.

## Introduction

Over the last two decades, scholars have paid increasing attention to those with callings as viewing work as a calling has been found to be beneficial to individuals and organizations (Wrzesniewski et al., 1997; Bunderson and Thompson, 2009; Duffy et al., 2011). Experiencing a calling positively associates with work, career, and life outcomes across social status (Duffy and Autin, 2013), occupation types (Wrzesniewski et al., 1997), and cultures (Hagmaier and Abele, 2012; Park et al., 2016; Xie et al., 2016). Viewing work as a calling is related to greater job satisfaction, career commitment (Duffy et al., 2011), and goal-directed behaviors at work (Praskova et al., 2014). Although some scholars suggest that calling is primarily perceived through work itself and that it can be decoupled from membership in an organization (Wrzesniewski, 2012), experiencing a calling also relates to positive attitudes and behaviors toward colleagues (Bunderson and Thompson, 2009) and organizations (Cardador et al., 2011; Xie et al., 2017). Those who viewed their work as a calling exhibited more helping behavior toward coworkers (Bunderson and Thompson, 2009; Park et al., 2016) and organizations (Xie et al., 2017) and higher organizational attachment (Cardador et al., 2011) than those who did not.

In spite of the interpersonal and organizational benefits of callings, most calling research has focused on how an individual’s own calling influences those who endorse it; in contrast, whether people are influenced by another’s calling has been understudied. Do the effects of experiencing a calling extend beyond the realm of self? If so, how and to what extent are people influenced by others’ callings? This study aims to answer such questions by studying leader’s calling and its effects on followers’ attitudes and behaviors.

Interpersonal and social characteristics are critical components of motivational, attitudinal, and behavioral outcomes at work (Grant, 2007; Humphrey et al., 2007), and interaction with leaders has been recognized as an influential factor in shaping and promoting followers’ positive work attitudes (Humphrey et al., 2007; Ng and Feldman, 2014). Leaders raise the salience of certain values to achieve their goals (Bass, 1985; Shamir et al., 1993), and leaders’ implicit values and explicit behaviors and expressions of emotion are perceived by and contagious for followers (Cherulnik et al., 2001; Sy et al., 2005; Fu et al., 2010). Given that people with callings have differential behavioral, attitudinal, and emotional effects from people with job or career work orientations (Wrzesniewski et al., 1997) and there exist prevalent effects of calling at work (Bunderson and Thompson, 2009; Duffy and Dik, 2013; Schabram and Maitlis, 2017), leader’s calling is likely to impact followers both directly and indirectly through leaders’ thoughts, emotions, and behaviors.

The primary goal of this study is to examine the assumption that leader’s calling would have positive effects on three sets of follower outcomes: team commitment, voice behavior, and job performance. By examining both an attitudinal variable (team commitment) and two behavioral variables (voice behavior and job performance), we aimed to show that leader’s calling can affect not only followers’ attitudes, but also actual behaviors. In particular, there has been a call to examine more closely the relationships between calling and in-role and extra-role behaviors (Duffy and Dik, 2013). Despite its importance and increasing attention in psychology and management fields (LePine and Van Dyne, 1998; Morrison, 2014), voice behavior—a form of extra-role behavior that emphasizes expression of constructive challenge to improve situations (Van Dyne and LePine, 1998)—has received little attention in calling literature. The second goal of this study is to examine a mediating variable through which leader’s calling influences followers’ team commitment, voice behavior, and job performance by focusing on follower perceptions of transformational leadership. Given that calling is action-oriented (Elangovan et al., 2010), leaders who experience a calling are likely to express and live out their callings. We propose that transformational behaviors that are aligned with the characteristics of calling would mediate the relationships between leader’s calling and the three follower outcomes.

To explore the role of leader’s calling on followers, we elected to study a sample of leaders and followers in the Republic of Korea Air Force (ROKAF). Given that soldiers engage in prosocial occupations to protect their country and sacrifice self-interest to do so, those with strong callings are likely to join the military, and a leader’s calling can be perceived and is observable to followers. To our knowledge, this is the first study to examine the effects of leader’s calling, and prosocial jobs that have been widely studied in calling literature (e.g., Bunderson and Thompson, 2009; Cardador et al., 2011; Schabram and Maitlis, 2017) can serve as effective and beneficial samples to address questions about leader’s calling. Also, in military settings (Dvir et al., 2002; Bass et al., 2003) and aircrew teams (Chidester et al., 1991), the role of leader has been found to be critical in facilitating followers’ commitment, communication, and performance. For flight crews in both the military and private sectors, how leaders communicate, coordinate with crew members, and make decisions during the flight have been shown to have decisive influences on flight performance and safety behaviors (Chidester et al., 1991; Merritt, 2000). In particular, in an East Asian cultural context—in which power distance is high—the leader’s role has been revealed as even more important, because followers are unlikely to express opinions that conflict with the leader’s views or to freely exchange ideas unless the leader actively listens to followers and encourages communication within a team (Helmreich et al., 2001). We believed that our sample would be useful in identifying the distinctive role of leader’s calling.

### Leader’s Calling

Although there is no consensus on how to conceptualize a calling, scholars commonly describe a calling as work that is meaningful (Wrzesniewski, 2003; Dobrow and Tosti-Kharas, 2011; Dik et al., 2012; Hagmaier and Abele, 2012;) and that is driven by external forces (Dik et al., 2012; Hagmaier and Abele, 2012). Strong meaningfulness and the sense of a guiding force urge people with callings to act on their callings despite obstacles and unexpected challenges (Elangovan et al., 2010; Wrzesniewski, 2012; Schabram and Maitlis, 2017). When people perceive a calling, they try to live out their callings and enact them by improving their skills and pursuing the careers they feel called to (Dobrow-Riza and Heller, 2015; Schabram and Maitlis, 2017). Those who experience a calling report greater levels of motivational resources, such as intrinsic motivation (Conway et al., 2015) and psychological capital (Choi et al., in press), which promotes their well-being (Conway et al., 2015). Such accumulated psychological resources and increased satisfaction with their jobs and lives, in turn, contribute to strengthen and maintain their callings (Duffy et al., 2014; Conway et al., 2015; Schabram and Maitlis, 2017). In particular, leaders who typically possess formal status and power (Magee and Galinsky, 2008) were found to feel relatively autonomous in living out their calling (Hirschi et al., 2018). Leaders who view their work as a calling are likely to express and act on their calling based on personal and job resources—and, as a result, the endorsement of calling would influence others at work.

Experiencing a calling not only benefits those who experience it, but also colleagues, teams, and organizations (Wrzesniewski, 2003; Bunderson and Thompson, 2009; Cardador et al., 2011). People with callings identify themselves with their occupation, community, and organization by embracing the beliefs and values of their work (Bunderson and Thompson, 2009; Cardador et al., 2011). They define their workplace as a central part of the self (Bunderson and Thompson, 2009) and believe that their organization is integral to their goal fulfillment (Cardador et al., 2011). This strong identification with and passion for work motivate people to commit to their work and organizations (Cardador et al., 2011; Duffy et al., 2011; Xie et al., 2017), help others (Park et al., 2016), and devote extra time to work (Bunderson and Thompson, 2009). In addition to the positive interpersonal and organizational effects of calling, Wrzesniewski (2003) asserts that calling may play a role in work-group functioning and that combinatory effects of work orientation may exist within work groups. She supports this argument by demonstrating that teams with a higher proportion of members with calling orientation reported higher levels of team identification, commitment, and trust in management than teams with a smaller proportion of members with callings (Wrzesniewski, 2003). Such findings suggest that the beneficial effects of a leader’s calling endorsement can extend beyond the individual and affect team members. In this study, we examined the effects of leader’s calling on followers’ team commitment and two types of performance, voice behavior and job performance.

### Leader’s Calling and Follower’s Team Commitment, Voice Behavior, and Job Performance

As an affective response to a team, team commitment refers to the degree to which an individual identifies with a team and attaches to a team to achieve their goals (Mowday et al., 1979). Social exchange theory suggests that social exchanges involve unspecified obligations assumed by individuals in response to favors they have received (Blau, 1964). When employees receive fair and equitable treatment from leaders, their perceptions of fairness increase their commitment to teams and organizations (Bishop and Scott, 2000). People with callings have been shown to define their job as a moral duty, elicit ethical behaviors, and spend extra hours at work to meet high moral standards (Bunderson and Thompson, 2009). Followers would show high team commitment when they experience conscientious and ethical behaviors by leaders who view their work as a calling. Also, followers’ social membership increases their team commitment via frequent interactions within a team (Pratt and Ashforth, 2003). People who perceive a sense of calling and work meaningfulness exert efforts to strengthen a sense of community and kinship to achieve their work goals (Ashforth and Kreiner, 1999; Bunderson and Thompson, 2009). A leader who views their work as a calling is likely to promote followers’ sense of connectedness at work and team commitment to achieve their goals. Consistent with this assumption, teams with a high proportion of people with calling orientation engaged in frequent communication and displayed high team commitment (Wrzesniewski, 2003). These findings lead us to propose a positive association between leader’s calling and follower team commitment.

Voice behavior refers to the extent to which individuals speak out and challenge the status quo to improve situations (Van Dyne and LePine, 1998). Research on voice behaviors has emphasized the role of leaders as situational facilitators (LePine and Van Dyne, 1998; Detert and Burris, 2007). Certain types of leader attitudes and behaviors have been found to create and promote psychological safety, which is a major psychological condition that predicts follower voice behavior (Detert and Burris, 2007; Morrison, 2014). In a psychologically safe environment, followers express their opinions and offer constructive suggestions without worrying about potential negative feedback from supervisors and others (Detert and Burris, 2007; Liang et al., 2012). Also, studies have found that leader inclusiveness, which invites and appreciates followers’ contributions in a team (Nembhard and Edmondson, 2006) and a leader’s relational approach, which focuses on quality relationship building with team members (Carmeli and Gittell, 2009), enhanced followers’ voice behaviors through increased psychological safety. Scholars have demonstrated that leaders who view their work as a calling provide constructive feedback to followers (Schabram and Maitlis, 2017), are perceived as highly connected with others at work (Cardador et al., 2011), and are likely to have a high level of inclusiveness and to promote voice behaviors (Nembhard and Edmondson, 2006). Given that quality relationships are a major channel to promote one’s meaningfulness (Pratt and Ashforth, 2003), experiencing a calling partly stems from quality relationships with others at work (Dobrow, 2013); in turn, it strengthens relationship quality (Bunderson and Thompson, 2009). A leader with a calling is likely to promote followers’ voice behavior based on a strong sense of connectedness and increased psychological safety.

Follower job performance is also likely to be influenced by leader’s calling. How leaders view and communicate work impacts followers’ job performance by changing their perceptions of core characteristics (Piccolo and Colquitt, 2006). When leaders experience a calling, they are likely to have a clear sense of purpose and meaning at work (Hall and Chandler, 2005; Dik and Duffy, 2009; Elangovan et al., 2010) and to focus on the prosocial nature of work (Dik and Duffy, 2009; Elangovan et al., 2010). A leader’s clear vision and prosocial perspective on how their work influences others increase followers’ work meaningfulness and job performance (Arnold et al., 2007; Grant, 2012). Qualitative research on calling also lends support for the positive influence of leader’s calling on follower performance. Using retrospective narrative interviews, Schabram and Maitlis (2017) found that workers with callings did not differ in terms of talent or ability upon career entry, but they eventually performed better and led their teams to success when they were in a managerial position, due to their accumulated skills and knowledge. Based on these findings, we expected that leader’s calling would be positively associated with follower job performance.

**Hypothesis 1:** Leader’s calling is positively related to follower team commitment (1a), follower voice behavior (1b), and follower job performance (1c).

### The Role of Transformational Leadership

We propose that one plausible mechanism through which leader’s calling may have positive effects on followers’ commitment, voice behavior, and job performance is follower perception of transformational leadership. A sense of calling reflects people’s relation to their work and people with callings view their work as a vocation to fulfill their values and meaning (Wrzesniewski et al., 1997). According to a career calling model (Hall and Chandler, 2005), a sense of calling can be understood through goal-setting perspective and motivation theory (Duffy et al., 2017). People with a calling develop a strong focus on the goals that reflect their purpose, and become effortful in achieving their calling (Hall and Chandler, 2005). Their efforts motivate people with a calling to increase efforts and goal-oriented behaviors (Praskova et al., 2014). In a similar vein, the effects of transformational leadership on followers are explained by self-concordance theory (Bono and Judge, 2003)—a psychological theory of motivation and self-regulation (Sheldon and Elliot, 1999). According to self-concordance theory (Sheldon and Elliot, 1999), goals that are consistent with one’s values and interests lead to goal attainment and well-being. The goal setting and self-concordance theories can be used as a overarching theory of this study. The theories suggest that leader’s calling may promote transformational behaviors that facilitate their calling and goals, and transformational leader behaviors motivate followers to commitment and perform better based on their self-congruent goals and values (Bono and Judge, 2003). Although the empirical and theoretical relations between leader’s calling and transformational leadership have received little attention, qualitative research on calling (Bunderson and Thompson, 2009; Schabram and Maitlis, 2017) and other research that supports conceptual links on the study variables suggest that transformational leadership serve as a mediator on the links between leader’s calling and the three outcomes.

Bass (1985) characterized transformational leadership as encompassing four distinct components: inspirational motivation, idealized influence, individualized consideration, and intellectual stimulation. Leaders with inspirational motivation articulate attractive visions and demonstrate optimism and enthusiasm. Idealized influence is the degree to which leaders place followers’ needs over their own and demonstrate high ethical standards. Individual consideration involves providing support and mentoring for followers and paying attention to their needs for growth and achievement. The last component, intellectual stimulation, is the degree to which leaders encourage followers to question assumptions and approach old situations with new perspectives.

Transformational leaders influence followers through changes in follower perceptions about jobs (Piccolo and Colquitt, 2006) as well as changes in follower self-concepts (Shamir et al., 1993). Leaders frame followers’ work experiences for better understanding and make meanings out of their work and work environment (Smircich and Morgan, 1982). Studies show that transformational leaders play a particularly strong role in making meanings and imbuing work with meaningfulness for followers (Arnold et al., 2007) by articulating a clear and meaningful vision, emphasizing collective identities, and referencing core job characteristics (Bass, 1985; Shamir et al., 1993; Piccolo and Colquitt, 2006). Given that calling is deeply rooted in one’s identity and purpose, people with callings attend to and actively frame social cues in work environment (Schabram and Maitlis, 2017). Strong goals and belief in one’s work sometimes cause people with callings to experience confusion about and conflicts between their values and reality (Bunderson and Thompson, 2009), and active meaning-making and proactive adjustment to situations are required to maintain their callings (Schabram and Maitlis, 2017). Through intense interpretation and subsequent actions to make and maintain meanings, people with callings learn how to frame their work and adapt to inner and situational conflicts (Schabram and Maitlis, 2017). Such findings suggest that leaders who view their work as a calling are likely to help followers to make meanings in their own work and bucket their work in a meaningful way; such attitudes and behaviors are consistent with those of transformational leadership.

Also, people with callings show high intrinsic motivation that forms the basis of transformational leader behaviors. When people experience a calling, their experiences generate more intrinsic motivation and identified motivation (Conway et al., 2015). This causal direction from experiencing a calling to intrinsic and identified motivation was revealed in a diary study (Conway et al., 2015). Researchers have also found that a manager’s calling is positively associated with intrinsic motivation (Dobrow and Tosti-Kharas, 2011). Research that investigates individual predictors of transformational leadership shows that intrinsic motivation and a desire to achieve an ideal self precede transformational leader behaviors (Barbuto, 2005). Given that calling urges people to achieve an ideal self and to reduce discrepancies between the ideal self, actual self, and ought self (Elangovan et al., 2010), leaders with callings are motivated to reach an ideal self and exhibit more transformational behaviors.

In addition, many of the behaviors elicited by calling endorsement have direct implications for transformational leader behaviors. People with callings are clear about their goals and desired states (Hall and Chandler, 2005; Elangovan et al., 2010), and this confidence may lead to more behaviors that articulate a vision for followers. Those who perceive their work as a calling emphasize the ethical consequences of their work (Bunderson and Thompson, 2009) and believe that their work contributes to the collective good (Dik and Duffy, 2009), and therefore may engage in behaviors of idealized influence. Leaders with callings were highly interested in developing followers (Schabram and Maitlis, 2017), which is likely to motivate more individualized considerate behaviors. Also, people with callings who focus on developing their abilities and skills (Schabram and Maitlis, 2017; Lysova et al., 2018) may intellectually stimulate their followers. Building on these findings, we hypothesized that:

**Hypothesis 2:** Leader’s calling is positively related to follower perceptions of transformational leadership.

The positive effects of transformational leadership on followers’ team commitment, voice behavior, and job performance have received consistent support in leadership literature. Transformational leaders promote followers’ commitment by encouraging them to take novel approaches to their work (Bass, 1985), realigning followers’ personal values with the leader’s goals, and internalizing these values (Jung and Avolio, 2000). In a longitudinal study that explored the development of a team and interactions within a team, leaders’ transformational behaviors promoted team commitment based on shared vision and increased interdependence among team members (Lorinkova et al., 2013). This positive linkage between transformational leadership and commitment was also found in samples of military leaders (Bass et al., 2003) and East Asians (Fu et al., 2010).

According to a review of voice behavior (Morrison, 2014), leaders play an important role in predicting employees’ voice behavior because they provide cues to subordinates on whether speaking up is valued and safe. By definition, transformational leaders intellectually stimulate followers to view situations with novel perspectives and change the status quo (Avolio et al., 1999). Transformational leaders’ idealized influence and individual consideration also encourage followers to speak up by fostering their psychological safety (Detert and Burris, 2007) and promoting organizational and personal identification (Liu et al., 2010). Empirical research demonstrates that transformational leadership is positively related to employees’ voice behavior across cultures (Detert and Burris, 2007; Liu et al., 2010; Conchie et al., 2012).

Meta-analyses have found that transformational leadership is positively related to job performance in different types of organizations across cultures (Fuller et al., 1996; Judge and Piccolo, 2004), and transformational leaders promote follower job performance through diverse psychological and behavioral mechanisms. Transformational leaders who emphasize the development and growth of followers provide constructive feedback to their followers and encourage them to think creatively (Bass, 1985). Based on their leader’s feedback, accumulated novel approaches, and successful experiences at work, followers become more self-efficacious and eventually perform better (Bandura, 1977). Also, followers’ social and personal identification is enhanced by a transformational leader’s behaviors (Kark et al., 2003; Wang et al., 2005), which increases the leader’s recognition and followers’ trust in the leader (Jung and Avolio, 2000) and, in turn, bolsters followers’ self-efficacy and job performance (Kark et al., 2003; Wang et al., 2005). A positive causal influence of transformational leadership on job performance has been demonstrated (Jung and Avolio, 2000; Dvir et al., 2002), and the positive associations between military leaders’ transformational leadership and task performance have received support (Shamir et al., 1993; Dvir et al., 2002; Bass et al., 2003). Based on such findings, we suggest that one route by which leader’s calling may influence follower commitment, voice behavior, and job performance is through followers’ perceived transformational leadership.

**Hypothesis 3:** Follower perceptions of transformational leadership partly mediate the relationships between leader’s calling and follower team commitment (3a), follower voice behavior (3b), and follower job performance (3c).

## Materials and Methods

### Participants and Procedures

The sample consisted of 284 matched pairs of combat flight leaders and followers in the Republic of Korea Air Force. One leader and one follower composed a team, and the temporary team was designed to fly a combat aircraft once and dismissed after the training. The team is only designed for the flight training. The leader and follower in a team were acquainted with each other, as they belonged to the same fighter squadron, but their relationship was not based on consistent or frequent interaction. The leader’s role was to lead the pre-meetings, help the follower make decisions during the flight, conduct the debriefing on flight performance, and assess flight performance. The role of the follower was to take part in the pre-meetings and the debriefing session, and perform the flight tasks.

The team conducted three meetings: two pre-flight meetings and a debriefing meeting. At the first pre-flight meeting, a leader explains mission for the flight, sets a goal for follower, and discusses how to allocate tasks the day before the flight. At the second flight meeting, which is held 2 or 3 h before the flight, a leader gives specific directions for the flight along with weather condition and current obstacles. The last meeting, a debriefing meeting, is held after the flight. In the meeting, a leader debriefs the flight focusing on the degree to which goals and missions are accomplished and how to improve follower’s ability and skills based on the flight. Each meeting takes about 2 or 3 h and the flight takes averages one to one and a half hours.

Prior to data collection, we received approval from the Republic of Korea Air Force headquarters and were provided available survey dates based on combat flight training schedules. On flight training days, we visited the Republic of Korea Air Force wings that were stationed in six cities in South Korea. After flight training and debriefing were completed, we approached those who were finished debriefing, explained our research objectives, and asked them to participate in the survey on a voluntary basis. Those who completed a consent form took a paper-and-pencil survey.

The survey was conducted in Korean, and all variables used in the study had been translated and validated in Korean. Leaders rated their own level of calling, follower voice behavior, and job performance. Followers rated their perceptions of transformational leadership and team commitment. Demographic variables such as gender, age, and tenure were self-reported. 289 matched pairs completed the flight training session and a total of 284 independent matched pairs participated the survey with a response rate of 98.26%. Participants were predominantly male (leaders, 97.91%; followers, 98.62%), and the average age for leaders was 32.04 years (*SD* = 3.21) and for followers was 27.60 years (*SD* = 2.51). Average tenure for leaders was 91.27 months (*SD* = 33.84), and 55.80 months (*SD* = 40.55) for followers.

### Instruments

#### Calling

We measured calling with a Multidimensional Calling Measure (MCM) developed by Hagmaier and Abele (2012). The nine-item scale consists of three sub dimensions: identification/person-environment fit (IP), transcendent guiding force (TGF), and sense and meaning and value-driven behavior (SMVD). Participants rated the extent to which they viewed their work as a calling using a 5-point Likert scale (1 = *not all true of me*, 5 = *totally true of me*). Sample items include “I identify with my work” and “By doing my job, we serve the common good.” In this study, internal consistency reliability for calling was α = 0.82.

#### Transformational Leadership

We measured transformational leader behaviors with 16 items of Bass and Avolio’s Multifactor Leadership Questionnaire 5X (MLQ; Avolio et al., 1999), which included the four behavioral components: inspirational motivation (IM), idealized influence (II), individualized consideration (IC), and intellectual stimulation (IS). Followers completed a total of 16 items on a 5-point Likert scale (1 = *not at all* to 5 = *very likely*). Sample items include “My leader articulates a compelling vision of the future” and “My leader treats me as an individual rather than just a member of a group.” In this study, internal consistency reliability for the scale was α = 0.94.

#### Team Commitment

We measured follower team commitment with four items based on Mowday et al. (1979) organizational commitment questionnaire and Seashore’s (1979) cohesiveness measure. The scale was adapted from a prior study to measure team commitment (Van Der Vegt et al., 2000). Followers responded to four items on a 5-point Liker scale (1 = *strongly disagree* to 5 = *strongly agree*): “I feel proud to belong to this team,” “I feel very committed to this team,” “I feel very committed to this team,” and “I am willing to exert extra effort to help this team succeed.” In this study, internal consistency reliability was α = 0.84.

#### Voice Behavior

We assessed follower voice behavior with the five items developed by Van Dyne et al. (2003). Based on employee motives, Van Dyne et al. (2003) suggest three types of voice behaviors: acquiescent voice, defensive voice, and prosocial voice. In this study, we used five items of prosocial voice to measure the extent to which employees express work-related ideas or opinions based on cooperative motives, because this is a proactive and other-oriented form of voice behavior that aligns more closely with definitions of voice used by other scholars (LePine and Van Dyne, 1998; Nembhard and Edmondson, 2006; Morrison, 2014). Leaders rated follower voice behavior on a 5-point Likert scale (1 = *strongly disagree* to 5 = *strongly agree*). A sample item includes “This follower suggests ideas for change, based on constructive concern for the organization.” In this study, internal consistency reliability was α = 0.95.

#### Job Performance

We measured follower job performance with four items developed by Pearce and Porter (1986) that have been used in other research (Hochwarter et al., 1999). Leaders rated follower job performance along four dimensions: overall performance, completing tasks on time, quality of team performance, and achievement of work goals. A sample item includes “This follower completes work goals during flight.” Job performance was assessed on a 5-point Likert scale (1 = *very poor* to 5 = *outstanding*). In this study, internal consistency reliability was α = 0.90.

## Results

First, we conducted a confirmatory factor analysis in Amos 18 to examine the convergent and discriminant validity of study variables. We examined the five-factor model (e.g., leader’s calling, transformational leadership, team commitment, job performance, voice behavior) fit that corresponded to our predictions. The five-factor model provided an excellent fit to the data, χ^2^ = 215.63, *df* = 160, *p* < 0.01, CFI = 0.99, IFI = 0.98, RMSEA = 0.04. We then examined model fit for the four-factor model, which combines two leader-rated job behaviors, voice behavior, and job performance, χ^2^ = 899.13, *df* = 164, *p* < 0.01, CFI = 0.81, IFI = 0.81, RMSEA = 0.13, and the two-factor model, which combines the variables rated by leaders (leader’s calling, follower voice behavior, job performance) and the variables rated by followers (follower team commitment, transformational leadership), χ^2^ = 1244.77, *df* = 169, *p* < 0.01, CFI = 0.72, IFI = 0.72, RMSEA = 0.15. Lastly, we examined fit indices of one factor model that combines all the study variables as a factor, χ^2^ = 2795.92, *df* = 230, *p* < 0.01, CFI = 0.40, IFI = 0.40, RMSEA = 0.20. To determine whether the five-factor model represented better fit than other models, we calculated differences in chi squares between the six-factor model and the other models. Results indicate that no other models showed better fit than the five-factor model.

We evaluate the magnitude of common method bias using two approaches. First, we examined Harman (1967)’s one-factor test. If a substantial amount of common method variance is present, a single factor will account for the majority of the covariance in the criterion variables (Podsakoff and Organ, 1986). The results revealed that seven distinct factors accounted for 65.82% of the total variance, with the first factor explaining 27.48%. Thus, no single factor emerged, nor did one factor account for most of the variance.

We also examined common method bias using the marker variable technique (Lindell and Whitney, 2001; Podsakoff et al., 2003, 2012). If a variable can be identified on theoretical grounds that should not be related to at least one other variables included in the study, then it can be used as a marker variable (Lindell and Whitney, 2001). Under the marker variable technique, common method variance was assessed based on correlations between the marker variable and research constructs because they are assumed to have no relationships (Lindell and Whitney, 2001; Podsakoff et al., 2012). Based on the findings that job tenure was not theoretically related to a sense of calling (Wrzesniewski et al., 1997), we used a job tenure as a marker variable. Except for follower’s voice behavior (*r* = -0.18, *p* < 0.01), job tenure was not significantly related to all substantive variables and the correlation coefficients ranged from 0.00 to 0.07. Recommended by Lindell and Whitney (2001), we used the partial correlation matrix derived from job tenure as an input in our path analysis model. The results showed neither the magnitude nor the significance of the coefficients of the indicators of interest changed substantively. The results suggested that this study is relatively robust against common method biases and provided support for the validity of our measures.

**Table 1** provides descriptive statistics and correlations for study variables. Consistent with our hypotheses, leader’s calling positively correlated with follower team commitment, follower voice behavior, and follower job performance. Follower perceptions of transformational leadership had positive correlations with leader’s calling, follower team commitment, and voice behavior, but the correlation between perceived transformational leadership and job performance was not significant. Among demographic variables, follower’s age and tenure positively correlated with follower voice behavior. Also, follower’s tenure was positively correlated with follower job performance. Thus, we controlled for follower’s age and tenure in analyzing structural equation modeling.

**Table 1:** Descriptive statistics and correlations for study variables (*N* = 284).

	Variable	Mean	*SD*	1	2	3	4	5	6
(1)	Follower age	27.60	2.51	–					
(2)	Follower tenure	55.80	40.55	0.56^∗∗^	–				
(3)	Leader’s calling	4.09	0.51	0.04^∗∗^	-0.02		–		
(4)	Transformational leadership	4.15	0.50	-0.12	-0.11	0.12^∗∗^	–		
(5)	Follower team commitment	4.33	0.56	-0.04	-0.01	0.18^∗∗^	0.68^∗∗^	–	
(6)	Follower voice behavior	3.44	0.88	0.18^∗∗^	0.14^∗^	0.23^∗∗^	0.19^∗∗^	0.18^∗∗^	–
(7)	Follower job performance	3.99	0.63	0.06^∗∗^	0.12^∗^	0.40^∗∗^	0.10^∗∗^	0.15^∗^	0.28^∗∗^

*^∗^p < 0.05, ^∗∗^p < 0.01*.

Parcels were created using a method proposed by Little et al. (2002). The three subscales of calling and the four subscales of transformational leadership were used as observed indicators. Because team commitment, voice behavior and job performance have less than six observed indicators, each observed variable was used as observed indicators.

To test our hypotheses, we proceeded to estimate a series of four contrasting models. We implemented a hypothesized partially mediated relationship (Model A), a fully mediated relationship between leader’s calling and the three outcomes (Model B), and two other models. Based on research in which the effect of transformational leadership on job performance was mediated by team commitment (Bass et al., 2003), and employees’ team commitment predicted their voice behaviors and job performance (Bishop and Scott, 2000), we included paths from team commitment to voice behaviors and job performance (Model C). Then, we specified that the last model (Model D), which posits that leader’s calling may directly relate to followers’ three outcomes, not be mediated by transformational leadership. We excluded a path from leader’s calling to transformational leadership to specify Model D. Differences between the models were assessed through the chi square difference test.

**Table 2** shows fit indices for the four models. The hypothesized partially mediated model (Model A) showed excellent fit in an absolute sense and provided better fit to the data than the fully mediated model (Model B), Δχ^2^(3) = 63.38, *p* < 0.01, Model C, Δχ^2^(1) = 5.36, *p* < 0.05, and Model D, Δχ^2^(1) = 4.76, *p* < 0.05. Thus, we chose the hypothesized model as our final model.

**Table 2:** Results of model comparison analyses.

	χ*^2^*	*df*	CFI	IFI	RMSEA	SRMR
A (Partially mediated)	259.94^∗∗^	197	0.98	0.99	0.03	0.05
B (Fully mediated)	323.32^∗∗^	200	0.97	0.97	0.05	0.10
C (Team commitment as a mediator)	265.30^∗∗^	196	0.98	0.98	0.04	0.05
D (Non-mediated)	264.70^∗∗^	198	0.98	0.98	0.04	0.06

*^∗∗^p < 0.01.*

**FIGURE 1:**
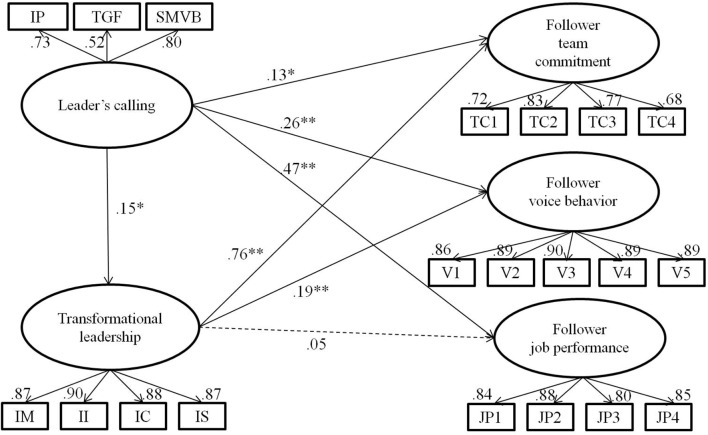
Standardized path estimates for the hypothesized model. ^∗∗^*p* < 0.01, ^∗^*p* < 0.05.

All parameters for the hypothesized model are displayed in **Figure 1**. Supporting Hypotheses 1a, 1b, and 1c, leader’s calling was positively related to followers’ team commitment (β = 0.13, *p* < 0.05), voice behavior (β = 0.26, *p* < 0.01), and job performance (β = 0.47, *p* < 0.01). Consistent with Hypothesis 2, leader’s calling was positively related to transformational leadership (β = 0.15, *p* < 0.05). In the hypothesized model, path estimates from follower perceptions of transformational leadership to team commitment (β = 0.76, *p* < 0.01) and to voice behavior (β = 0.19, *p* < 0.01) were significant, supporting a mediating role of transformational leadership on the links. Countering Hypothesis 3c, the relationship between transformational leadership and job performance was not significant (β = 0.05, *ns*), and the mediating role of transformational leadership on this relationship was not examined. To test the significance of indirect effects on the links from leader’s calling to follower commitment and voice behavior, we conducted 5,000 bootstrapping procedures recommended by Shrout and Bolger (2002). The indirect path from leader’s calling to follower team commitment was significant, 95% CI [0.01, 0.22], supporting Hypothesis 3a. The indirect path from leader’s calling to voice behavior was also significant, 95% CI [0.01, 0.07], which supports Hypothesis 3b.

## Discussion

In this study, we suggested and examined the effects of leader’s calling on followers. Our results support a direct and positive effect of leader’s calling on follower team commitment, voice behavior, and job performance. Our results provide insights into an extended role for calling by showing that employees are influenced by the extent to which others view their work as a calling, and the effects of calling can reach beyond those who endorse callings. Supporting a role of calling in diverse aspects of life, career, and job outcomes (Wrzesniewski et al., 1997; Duffy and Dik, 2013), leader’s calling is also positively associated with followers’ actual job behaviors, as well as with job attitude, which suggests the potentially wide influence of leader’s calling.

Our results show that leader’s calling is perceived by and transmitted to followers through follower perceptions of transformational leadership. The extent to which people view their work as a calling affects their behaviors (Duffy et al., 2011; Wrzesniewski, 2012), and the action-oriented nature of calling leads people to behave in certain ways to live out and act on their callings (Elangovan et al., 2010; Dobrow-Riza and Heller, 2015). The positive correlation between a leader’s self-rated calling and follower perceptions of transformational leadership suggests that leaders with callings demonstrate more transformational leader behaviors. The relationship between leader’s calling and transformational leadership is important, as transformational leader behavior predicts a host of positive job attitudes and outcomes at individual, team, and organizational levels (Fuller et al., 1996; Judge and Piccolo, 2004). Despite numerous studies that focus on the effects of transformational leadership, research regarding individual predictors of transformational leader behaviors has been limited (Judge and Bono, 2000; Barbuto, 2005). Our findings show that how leaders view their work and the degree to which they perceive their work as a calling can be a potential predictor of transformational leadership and leadership styles.

We found that the positive effects of leader’s calling on task commitment and voice behavior were in part due to follower perceptions of transformational leadership. This suggests that leader’s calling increases follower perceptions of transformational leadership, which in turn increases followers’ task commitment and voice behavior. However, the mediating role of transformational leadership on the relationship between leader’s calling and follower job performance was not supported, due to a non-significant association between transformational leadership and job performance. Despite considerable research that shows a positive association between transformational leadership and job performance (Judge and Piccolo, 2004), other research has found that transformational leaders do not always motivate follower job performance (Dvir et al., 2002; Bono and Judge, 2003). Dvir et al. (2002) found that platoon leaders’ transformational leadership did not influence follower job performance, and posited that followers’ focal interest may be performing better and getting better grades rather than developing themselves in stressful military-training settings. In this study, the stressful situation—in which a follower’s performance is evaluated after the flight task—is likely to motivate followers to focus more on how to receive a better evaluation for the task rather than how to develop knowledge and skills in the long run. Also, we assume that this non-significant relationship is in part due to common method bias, because leader’s calling and follower job performance were rated by a leader, while transformational leadership was rated by a follower.

### Theoretical and Practical Implications

This study contributes to advance previous research on calling by showing a sense of calling not only influences those who endorse a calling but those who work with them. Calling research, to date, has focused on individuals’ own calling and its effects of themselves. Although a sense of calling is found partially through a process of inner reflection or by a transcendental summons (Elangovan et al., 2010), interpersonal relations and social factors also have profound impact on formation and manifestation of a sense of calling and work meaningfulness (Rosso et al., 2010; Dobrow, 2013). Why people are influenced by other’s endorsement of calling can be partly due to an intuitive nature of a sense of calling. A strong sense of meaningfulness such as a sense of calling is associated with intuitive information processing or gut feelings (Heintzelman and King, 2016), and such quality has a ‘magnetic force’ in social interactions (Stillman et al., 2011). A strong sense of meaningfulness people with callings perceive is likely to be communicated to oneself and others in an intuitive manner, rather than through conscious reflection or a complicated cognitive process (Heintzelman and King, 2016). The results of this study shed light on the direct and indirect relations between leader’s calling and follower’s job attitudes and outcomes and it highlights the intuitive nature of a calling in interpersonal domains.

Our findings indicate that leader’s calling has direct positive influences on followers’ commitment, voice behavior, and job performance after accounting for the effects of transformational leadership. In particular, this study is based on South Korean combat pilots in a military setting, which is high power distance and collectivistic culture. Researchers proposed that the combination of high power distance and collectivistic culture among leaders is likely to negatively associate follower’s proactive behavior such as voice behaviors (Morrison and Milliken, 2000). Given leader’s calling motivates followers to enhance team commitment, voice behavior and job performance in a hierarchical and collectivistic culture, its beneficial role is expected to be observed in other organizational cultures.

On the practical perspective, organizations would be wise to support leader’s calling. A recent study on the relation between calling and job resources found that people with more job resources are likely to live their callings (Hirschi et al., 2018). Jobs that provide decision-making autonomy and task significance would be beneficial for leaders with callings to better live out their callings (Hirschi et al., 2018). Organizations and career counselors help leaders with callings to live out their calling by creating conditions that allow them to conduct more autonomous and meaningful tasks.

Given that perceived transformational leadership is a critical link from leader’s calling to follower’s commitment and to voice behaviors, training transformational behaviors to leaders and creating conditions to encourage transformational leadership is also helpful. Empirical research shows that transformational behaviors can be learned (Dvir et al., 2002). Mentoring and training programs might be helpful for developing transformational behaviors. Also, certain organizational cultures are more conductive to transformational leader behaviors (Pawar and Eastman, 1997; Phaneuf et al., 2016). For example, leaders who perceive their organizational cultures consistent with components of transformational leadership are more likely to engage in transformational behaviors (Phaneuf et al., 2016). Creating supportive and cooperative cultures that are consistent with components of transformational leadership can help facilitate the positive effects of leader’s calling on followers via enhanced transformational leader behaviors.

### Limitations and Future Research Directions

Several limitations of this study should be noted. First, while this study is based on dyadic data from leaders and followers—which may help alleviate concerns regarding common method bias to some degree—the cross-sectional study design cannot guarantee the causal directions among study variables. Future research using a longitudinal or quasi-experimental approach would likely clarify the effects of leader’s calling on followers’ team commitment, voice behavior, and job performance. Studies based on measures from other sources, such as colleagues, and conducted in a setting with two or more subordinates can help rule out this concern. However, in this study, only dyadic data were available and we assumed that the self-reported team commitment and supervisor-reported job performance and voice behavior were appropriate (Conway and Lance, 2010).

A second limitation concerns the study sample. A military setting in an East Asian culture is relatively hierarchical. Whether our findings can be generalized to other cultural contexts or types of organizational settings should be considered. In future research, additional contextual determinants could be integrated. Factors to be explored include organizational cultures, job characteristics, and perceived similarities between leaders and subordinates. However, given that the interaction between leader and follower is temporary, this study provides a conservative test of the effects of leader’s calling and how they are delivered to the follower. That is, the effects of a leader’s calling are expected to be observed and could be stronger in groups in which leaders are permanent. Therefore, we expect that the effects that occur in this setting would also occur in settings in which the leader’s role is more permanent.

Lastly, although we used a multidimensional measure of calling, it is not certain that it captures the nature of leader’s calling. For instance, some researchers define calling as a domain-specific construct, and suggest that a leader might perceive a calling in managerial work (Dobrow and Tosti-Kharas, 2011). Leaders in this study may perceive their calling as being a leader rather than a pilot. Changes in tasks and identities from non-manager to a manager might impact changes in the levels and nature of callings. More investigation of leader’s calling can deepen our understanding of the nature of leader’s calling and a sense of calling.

## Ethics Statement

This study was carried out in accordance with the recommendations of Yonsei University Institutional Review Board. The protocol was approved by the Yonsei University Institutional Review Board. All subjects gave written informed consent in accordance with the Declaration of Helsinki.

## Author Contributions

All authors designed the study. KL collected the data. JP performed the data analysis and wrote the manuscript, so worked as the first author. YS was the supervisor of the research team, and he repeatedly revised the manuscript with JP. All authors contributed to this work and approved the final version of the manuscript.

## Conflict of Interest Statement

The authors declare that the research was conducted in the absence of any commercial or financial relationships that could be construed as a potential conflict of interest.
